# Zanubrutinib plus R-CHOP improves the treatment effect of newly diagnosed diffuse large B cell lymphoma with double expression of MYC and BCL-2

**DOI:** 10.3389/fimmu.2025.1526318

**Published:** 2025-03-12

**Authors:** Min Zhang, Yingying Wu, Zhipeng Cheng, Lu Zhang, Lin Liu, Fang Liu, Guohui Cui, Linghui Xia, Yu Hu, Heng Mei, Tao Guo, Jun Fang

**Affiliations:** Institute of Hematology, Union Hospital, Tongji Medical College, Huazhong University of Science and Technology, Wuhan, China

**Keywords:** zanubrutinib, Bruton’s tyrosine kinase inhibitor, newly diagnosed DE-DLBCL, Ki67 index, p53 expression

## Abstract

**Background:**

Relevant studies have demonstrated the poor treatment outcomes and prognosis for double-expressor diffuse large B cell lymphoma (DE-DLBCL) in the rituximab era. Zanubrutinib plus R-CHOP (rituximab, cyclophosphamide, doxorubicin/liposomal doxorubicin, vincristine, prednisone; ZR-CHOP) has shown efficacy in untreated non-GCB DLBCL patients with extranodal involvement. However, its efficacy in newly diagnosed DE-DLBCL remains uncertain.

**Objective:**

This retrospective study sought to assess the efficacy and safety of ZR-CHOP in comparison to R-CHOP in treatment-naïve patients with DE-DLBCL.

**Method:**

This study assessed 78 patients with newly diagnosed DE-DLBCL who were admitted between June 2017 and January 2024. Among them, 55 patients received the R-CHOP regimen, while 23 patients were treated with the ZR-CHOP regimen. The clinical characteristics were well balanced between the two groups.

**Results:**

The complete response rates (CRR) were higher in the ZR-CHOP group than the R-CHOP group, regardless of whether patients completed 4 or 6 treatment cycles (P= 0.019; P= 0.025). ORR in the ZR-CHOP group showed a higher trend than that in the R-CHOP group (P= 0.624; P= 0.219). The median follow-up period was 23.3 months, and the predicted median progression free survival (PFS) in the R-CHOP group was 22.8 months, whereas the median PFS in the ZR-CHOP group was not reached. The 1-, 2-, and 3-year PFS rates in the ZR-CHOP group showed a beneficial trend compared with the R-CHOP group, but there was no statistical difference (P= 0.072). However, the PFS of the ZR-CHOP group was longer than that of the R-CHOP group in patients with Ki67 index >75% (P= 0.034) and p53 expression >50% (P= 0.0033). The predicted median overall survival (OS) in the ZR-CHOP and R-CHOP groups were not reached. The 1-, 2- and 3-year OS rates were not significantly different between the two groups (P= 0.29). The most common adverse event in both groups was hematotoxicity, but there was no significant difference in the incidence of all adverse events between the two groups.

**Conclusion:**

First-line treatment with the ZR-CHOP regimen improved CRR in the untreated patients with DE-DLBCL and prolonged PFS in the Ki67 index >75% subgroup and the p53 expression >50% subgroup.

## Introduction

Diffuse large B cell lymphoma (DLBCL) is the most common type of non-Hodgkin’s lymphoma and is characterized by high aggressiveness and heterogeneity (1). Double-expressor diffuse large B cell lymphoma (DE-DLBCL) co-expresses MYC and BCL-2 as determined by immunohistochemistry (IHC) (2, 3). The revised 2016 WHO classification recommends cutoff values of 40% for MYC and 50% for BCL-2 expression as assessed by IHC (4). MYC and BCL-2 overexpression is likely attributable to gene amplification and posttranslational processes in the absence of chromosomal translocations (5–7). DE-DLBCL accounts for 20-35% of new DLBCL cases. It is associated with an aggressive clinical course and is more common in the activated B-cell (ABC) subtype (8–10). DE-DLBCL has demonstrated distinctive clinical features such as older age, advanced Ann Arbor stage, higher lactate dehydrogenase (LDH) level, higher Ki67 proliferation index, and higher international prognostic index (11–13).

R-CHOP, an anthracycline-based regimen, is widely used as the first-line treatment for DLBCL (14–16). Multiple studies have identified the double-expressor status as an adverse prognostic factor for response to R-CHOP in DLBCL (9, 17). Relapsed/refractory DE-DLBCL patients often have inferior outcomes after autologous stem cell transplantation (ASCT) (18). Additionally, DE-DLBCL demonstrated a 10% risk of central nervous system (CNS) relapse at 2 years (19). These data highlight that DE-DLBCL is associated with adverse outcomes. Therefore, new treatment regimens with increased efficacy need to be developed for untreated DE-DLBCL patients to achieve better remission and long-term survival.

Bruton tyrosine kinase (BTK) is an essential component of the B-cell receptor intracellular signaling pathway, mediating B-cell development, proliferation, and survival (20). Recently, BTK inhibitors have proven to be a successful strategy for managing B-cell malignancies due to their broad efficacy across a range of diseases, safety, and the convenience of oral administration. The first-generation BTK inhibitor, ibrutinib, rapidly became the standard of care for treating patients with certain subtypes of non-Hodgkin lymphoma and chronic lymphocytic leukemia (CLL) (21–24). According to the latest results, event-free survival (EFS) of DE-DLBCL treated with ibrutinib combined with R-CHOP was superior to those receiving R-CHOP alone (25).

Zanubrutinib is a novel small molecule oral BTK inhibitor that effectively targets BTK (26). In a phase 1/2 clinical study, zanubrutinib demonstrated promising safety and efficacy in patients with relapsed/refractory DLBCL (27). In a phase 2 clinical study, the ZR-CHOP was found to be safe and effective for treating newly diagnosed non-GCB DLBCL patients with extranodal involvement (28). Another study indicated that non-GCB DLBCL patients with CD79B mutations, higher TCL1A expression, or high MYC/BCL-2 expression have clinical benefits to zanubrutinib monotherapy or combination therapy (29). At present, the prospective study of ZR-CHOP in the treatment of DE-DLBCL has attracted much attention, but the detailed data have not been officially published. The purpose of this retrospective study is to compare the effectiveness and safety of the ZR-CHOP regimen with the R-CHOP regimen in the treatment of newly diagnosed DE-DLBCL.

## Materials and methods

### Trial design and participants

This single-center, retrospective study evaluated the outcomes of patients with newly diagnosed DE-DLBCL who were treated with either the ZR-CHOP regimen or the R-CHOP regimen from June 2017 to January 2024, aiming to assess and compare the efficacy and safety of these two treatment protocols. The study was approved by the ethics committee and institutional review board of the Union Hospital Affiliated with Tongji Medical College, Huazhong University of Science and Technology (Ethics Approval No. UHCT240766). The study was conducted in compliance with the Declaration of Helsinki. As this study is retrospective and anonymous, informed consent was waived.

The inclusion criteria for patients were as follows: (1) no previous chemotherapy or targeted therapy; (2) pathologically confirmed DLBCL exhibiting co-expression of MYC (≥40%) and BCL2 (>50%) (2, 4); (3) availability of complete clinical and treatment information; (4) no involvement of central nervous system; (5) completion of at least four cycles of therapy.

### Treatment methods

Enrolled patients were divided into the ZR-CHOP regimen group and the R-CHOP regimen group. Zanubrutinib was administrated orally at a dose of 160 mg twice daily throughout the chemotherapeutic cycles. The R-CHOP regimen included both R-CHOP and R-miniCHOP. Some patients received liposomal doxorubicin instead of doxorubicin due to cardiac insufficiency, advanced age, and personal choice. The standard induction therapy for each group consisted of 6 cycles.

### Efficacy assessment

The evaluation of efficacy for nodular lymphoma was based on the Lugano 2014 criteria (30, 31). All patients were assessed for efficacy using enhanced computed tomography (CT) or positron emission tomography-computed tomography (PET/CT) scans. Prognostic stratification was conducted using the International Prognostic Index (IPI) and the National Comprehensive Cancer Network IPI (NCCN-IPI) (32, 33). The evaluation included complete response (CR), partial response (PR), stable disease (SD), and disease progression (PD) (34). The objective response rate (ORR) was calculated as the sum of the complete response rate (CRR) and partial response rate. Progression-free survival (PFS) was defined as the interval from diagnosis to the first occurrence of disease progression, death from any cause, or the last follow-up. Overall survival (OS) was defined as the duration from diagnosis to death or the last follow-up. Adverse events (AEs) were graded according to the National Cancer Institute Common Terminology Criteria for Adverse Events (version 5.0).

### Statistical methods

Data processing was carried out using Statistical Application System Software (SPSS) version 25.0 and R Studio version 4.4.1. Classification of case characteristics and treatment response rates were compared between groups using the “Tableone” package. OS and PFS were estimated using the Kaplan−Meier method and compared using the log-rank test. A P-value of <0.05 was considered statistically significant.

## Results

### Patient characteristics and treatment

**Figure 1:**
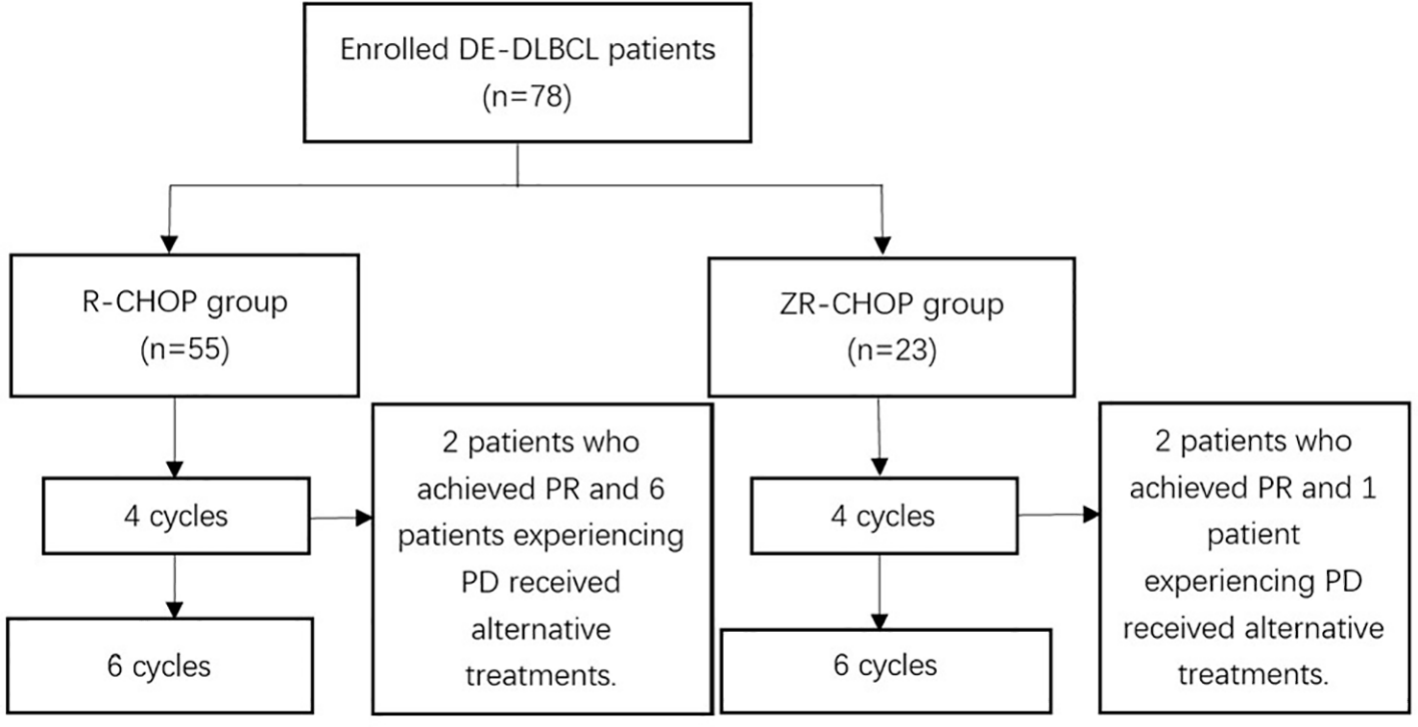
Treatment course of patients.

A total of 78 patients were included in the study, with a median follow-up of 23.3 months (range: 3.7–84.5 months) until May 2024. In the ZR-CHOP regimen group, two patients who achieved PR and one patient who experienced PD transitioned to alternative treatments during induction therapy. These treatments included zanubrutinib plus VR-DA-EPOCH (venetoclax, rituximab, dose-adjusted etoposide, prednisone, vincristine, doxorubicin and cyclophosphamide), zanubrutinib plus R-EPOCD (rituximab, etoposide, prednisone, vincristine, cyclophosphamide and liposomal doxorubicin), as well as zanubrutinib plus polatuzumab vedotin and zuberitamab (anti-CD20 antibody). In the R-CHOP regimen group, two patients achieving PR and six patients experiencing PD received alternative treatments, including R-GDP (rituximab with gemcitabine, dexamethasone, cisplatin), R-EPOCH (rituximab with etoposide, prednisone, vincristine, cyclophosphamide, doxorubicin), pola-R-CHP (polatuzumab vedotin plus rituximab, cyclophosphamide, doxorubicin and prednisolone), orelabrutinib plus R-CHOP and Chimeric antigen receptor (CAR)-T cell therapy (**Figure 1**).

There were no significant differences between the two groups, in term of age, gender, Ann Arbor staging, B symptoms, Eastern Cooperative Oncology Group (ECOG) score, LDH count, β2-microglobulin (β2-MG), IPI, NCCN-IPI, bone marrow involvement, extranodal involvement, bulky disease (≥7.5cm), cell of origin (Hans algorithm), expression of MYC and p53, as well as Ki67 index (**Table 1**).

**Table 1:** Baseline characteristics.

	R-CHOP (N=55)	ZR-CHOP (N=23)	P
Gender, n (%)			0.121
Female	26 (47.3)	16 (69.6)	
Male	29 (52.7)	7 (30.4)	
Age (years), median [range]	58.00 [27-76]	58.00 [24-72]	0.921
Ann Arbor staging system, n (%)			0.463
1	4 (7.3)	4 (17.4)	
2	18 (32.7)	7 (30.4)	
3	13 (23.6)	3 (13.0)	
4	20 (36.4)	9 (39.1)	
B symptoms, n (%)			0.643
No	36 (65.5)	17 (73.9)	
Yes	19 (34.5)	6 (26.1)	
ECOG score, n (%)			0.517
1	42 (76.4)	16 (69.6)	
2	10 (18.2)	4 (17.4)	
3	3 (5.5)	3 (13.0)	
LDH (U/L), median [IQR]	294.00[197.00, 564.00]	268.00[201.50, 438.00]	0.424
β2-MG (mg/L), median [IQR]	3.00 [2.45, 3.40]	2.80 [2.20, 3.45]	0.625
IPI, n (%)			0.948
0-1	16 (29.1)	6 (26.1)	
2	11 (20.0)	6 (26.1)	
3	18 (32.7)	7 (30.4)	
4-5	10 (18.2)	4 (17.4)	
NCCN-IPI, n (%)			0.739
0-1	6 (10.9)	3 (13.0)	
2-3	22 (40.0)	10 (43.5)	
4-5	23 (41.8)	7 (30.4)	
6-8	4 (7.3)	3 (13.0)	
Bone marrow involvement, n (%)			0.343
No	38 (69.1)	19 (82.6)	
Yes	17 (30.9)	4 (17.4)	
Extranodal involvement, n (%)			0.536
No	25 (45.5)	8 (34.8)	
Yes	30 (54.5)	15 (65.2)	
Bulky disease, n (%)			0.979
No	46 (83.6)	20 (87.0)	
Yes	9 (16.4)	3 (13.0)	
Ki67 index, median [IQR]	0.80 [0.70, 0.90]	0.80 [0.65, 0.88]	0.626
MYC expression, median [IQR]	0.50 [0.40, 0.60]	0.40 [0.40, 0.60]	0.307
p53 expression, median [IQR]	0.20 [0.05, 0.30]	0.20 [0.08, 0.60]	0.128
Cell of origin, n (%)			1
GCB	17 (30.9)	7 (30.4)	
non-GCB	38 (69.1)	16 (69.6)	

ECOG score, Eastern Cooperative Oncology Group Score; β2-MG, β2 microglobulin; IPI, International Prognostic Index; NCCN-IPI, National Comprehensive Cancer Network International Prognostic Index; GCB, germinal center B-cell-like lymphoma.

### Efficacy assessment

**Figure 2:**
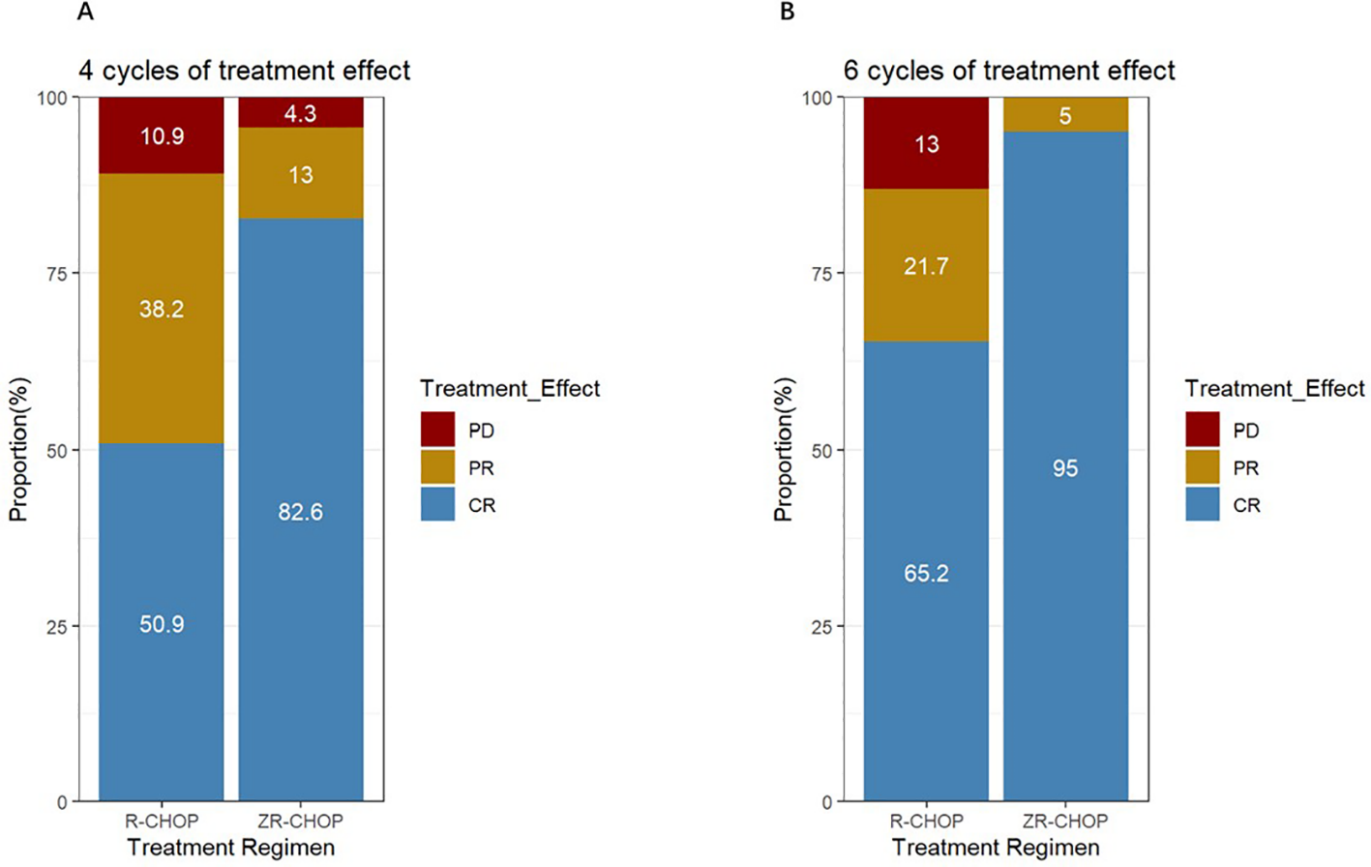
Comparisons of treatment efficacy between the two groups after 4 cycles **(A)** and 6 cycles **(B)** of treatment.

Twenty-three patients in the ZR-CHOP group and 55 patients in the R-CHOP group completed at least four cycles of therapy. The CRR was significantly higher in the ZR-CHOP group (82.6% vs. 50.9%, P = 0.019) (**Figure 2A**). After six cycles of treatment, twenty patients in the ZR-CHOP group and 46 patients in the R-CHOP group were evaluated. The CRR remained significantly higher in the ZR-CHOP regimen group (95% vs. 65.2%, P = 0.025) (**Figure 2B**). However, there was no statistically significant difference in ORR between the two groups (four cycles: 95.7% vs. 89.1%, P= 0.624; six cycles: 100% vs. 87%, P= 0.219).

**Figure 3:**
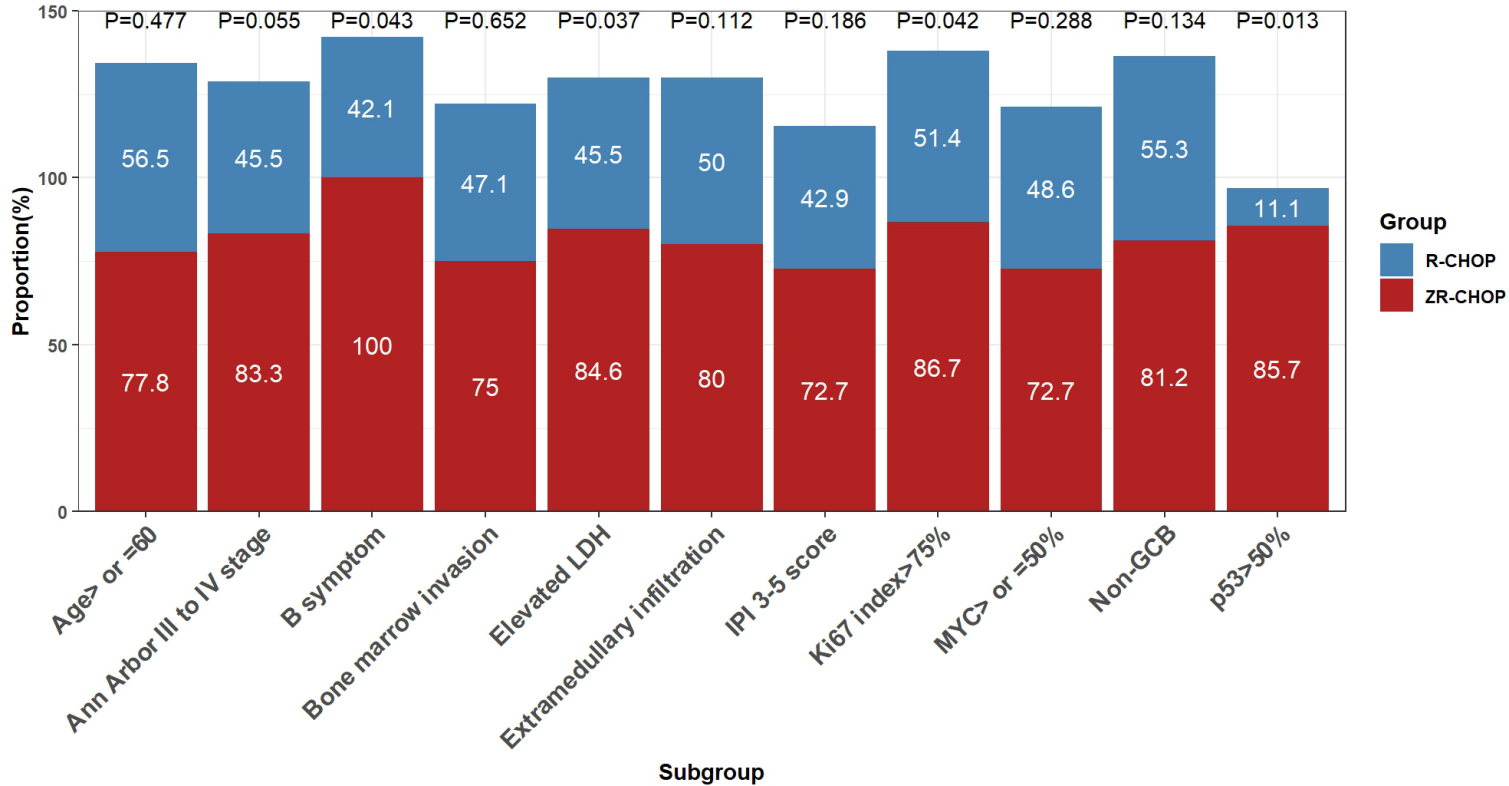
Comparisons of treatment efficacy among subgroups within the two groups after 4 cycles of therapy.

After four cycles of therapy, the complete response rates (CRRs) were significantly higher in the ZR-CHOP regimen group for patients with B symptoms (100% vs. 42.1%; P= 0.043), elevated LDH (84.6% vs. 45.5%; P= 0.037), p53 expression >50% (85.7% vs. 11.1%; P= 0.013), and Ki67 index >75% (86.7% vs. 51.4%; P= 0.042)(**Figure 3**). Additionally, the ZR-CHOP group exhibited a trend of higher CRRs in the other subgroups, including age ≥60 years (77.8% vs. 56.5%; P= 0.477), Ann Arbor stage of III and IV (83.3% vs. 45.5%; P= 0.055), bone marrow invasion (75% vs. 47.1%; P= 0.652), IPI score of 3-5 (72.7% vs. 42.9%; P= 0.186), extramedullary infiltration (80% vs. 50%; P= 0.112), MYC expression ≥50% (72.7% vs. 48.6%; P= 0.288), and non-GCB subtype (81.2% vs. 55.3%; P= 0.134).

**Figure 4:**
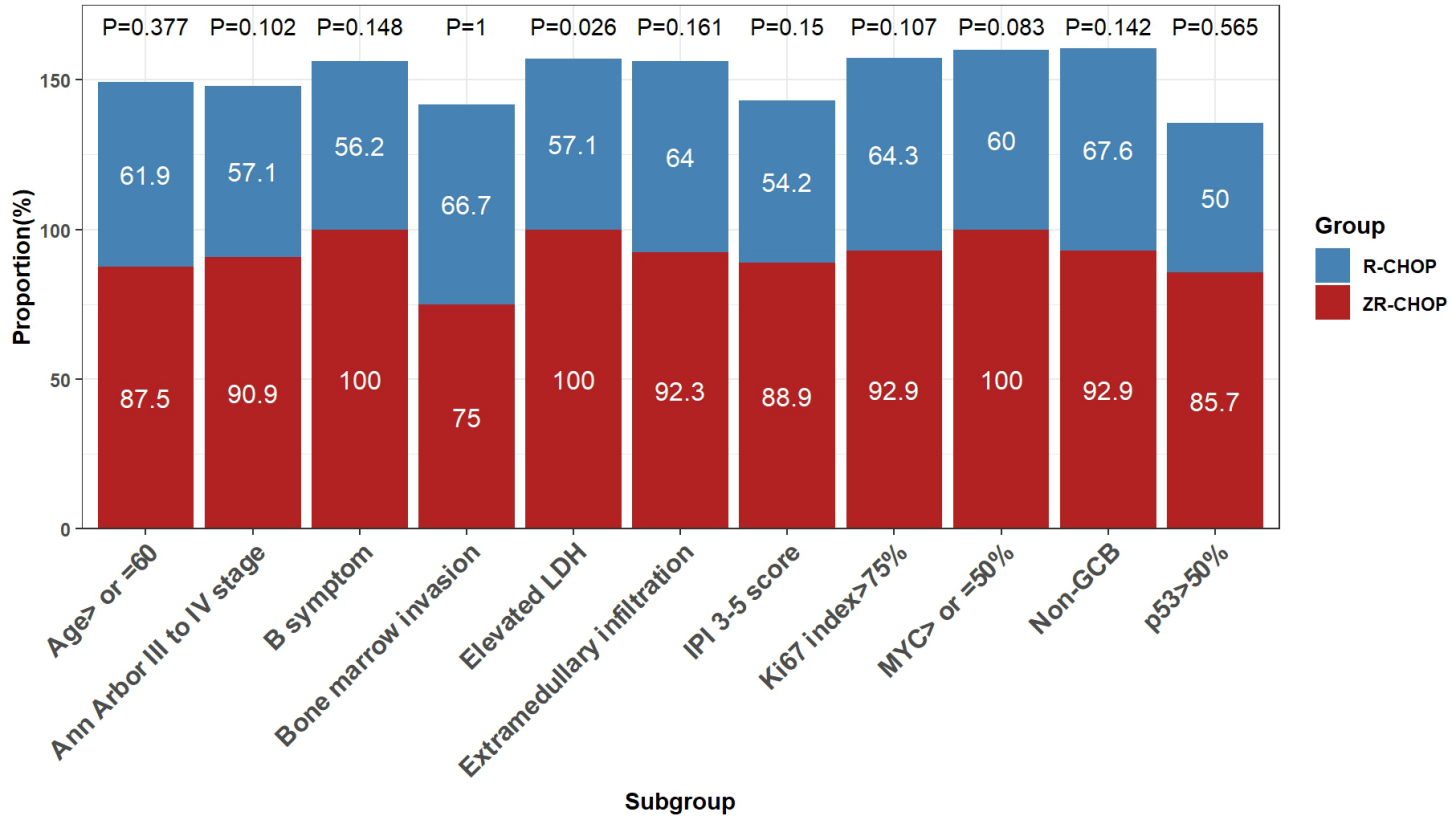
Comparisons of treatment efficacy among subgroups within the two groups after 6 cycles of therapy.

After six cycles of therapy, the CRRs were significantly higher in the ZR-CHOP regimen group for the elevated LDH subgroup (100% vs. 57.1%; P= 0.026) (**Figure 4**). Furthermore, there was a trend toward higher CRRs in certain subgroups within the ZR-CHOP group, including age ≥60 (87.5% vs. 61.9%; P= 0.377), Ann Arbor staging system III to IV (90.9% vs. 57.1%; P= 0.102), B symptoms (100% vs. 56.2%; P= 0.148), bone marrow invasion (75% vs. 66.7%; P= 1), IPI score of 3-5 (88.9% vs. 54.2%; P= 0.15), extramedullary infiltration (92.3% vs. 64%; P= 0.161), MYC expression ≥50% (100% vs. 60%; P= 0.083), p53 expression >50% (85.7% vs. 50%; P= 0.565), Ki67 index >75% (92.9% vs. 64.3%; P= 0.107) as well as non-GCB subtype (92.9% vs. 67.6%; P= 0.142).

**Figure 5:**
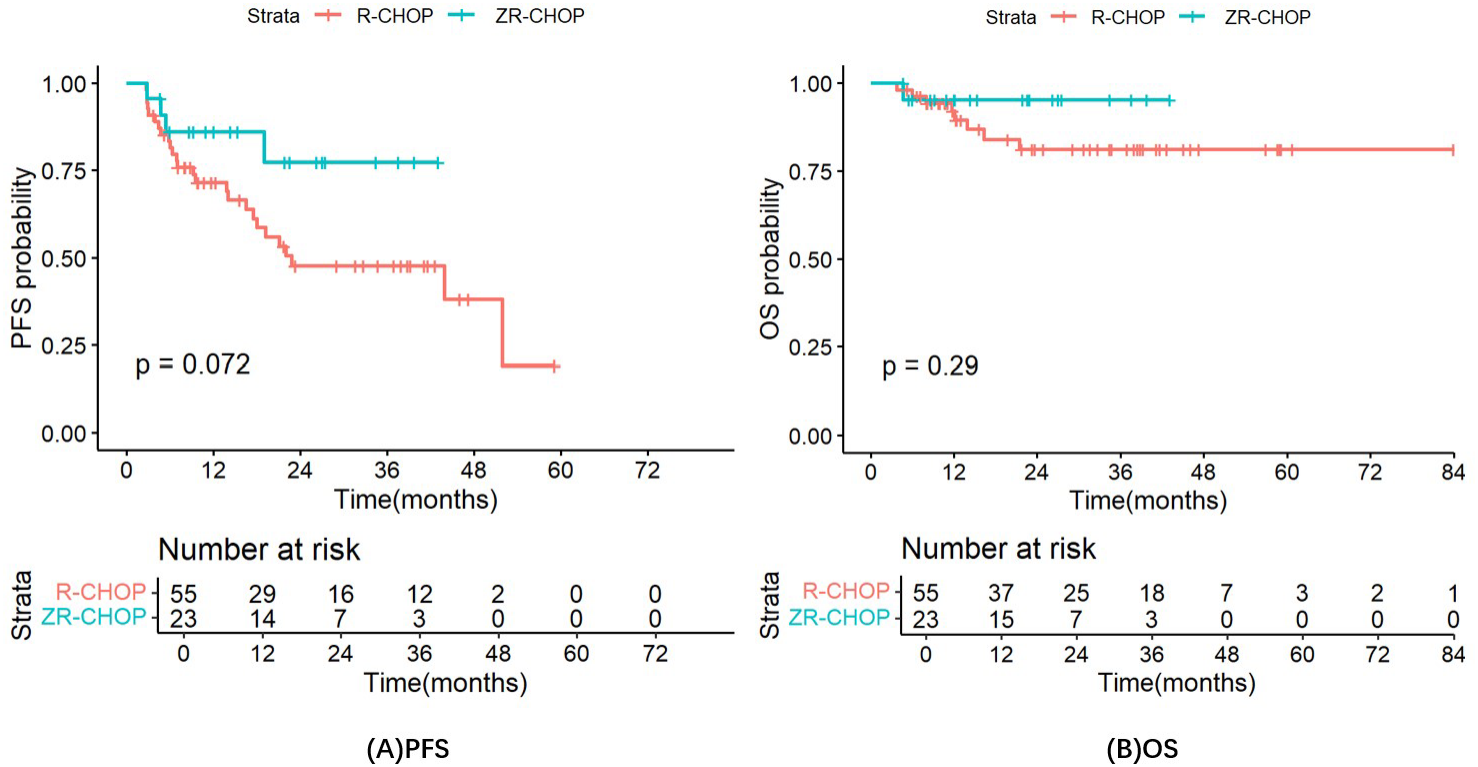
PFS **(A)** and OS **(B)** in the R-CHOP and ZR-CHOP regimen group.

In the ZR-CHOP regimen group, the 1-, 2- and 3-year PFS rates were 86.1%, 77.5%, and 77.5%, respectively, while the OS rates for these time points were consistently at 95.2%. In the R-CHOP regimen group, the PFS rates the 1, 2 and 3 year were 71.6%, 47.6%, and 47.6%, respectively, while the OS rates for these intervals were 92%, 81.1% and 81.1%, respectively. The predicted median PFS was 22.8 months in the R-CHOP group, whereas the median PFS was not reached in the ZR-CHOP group. Additionally, the predicted median OS was not reached in both groups. Although the PFS and OS were longer in the ZR-CHOP regimen group compared to the R-CHOP regimen group, the differences were not statistically significant (P= 0.072; P= 0.29) (**Figures 5A, B**).

**Figure 6:**
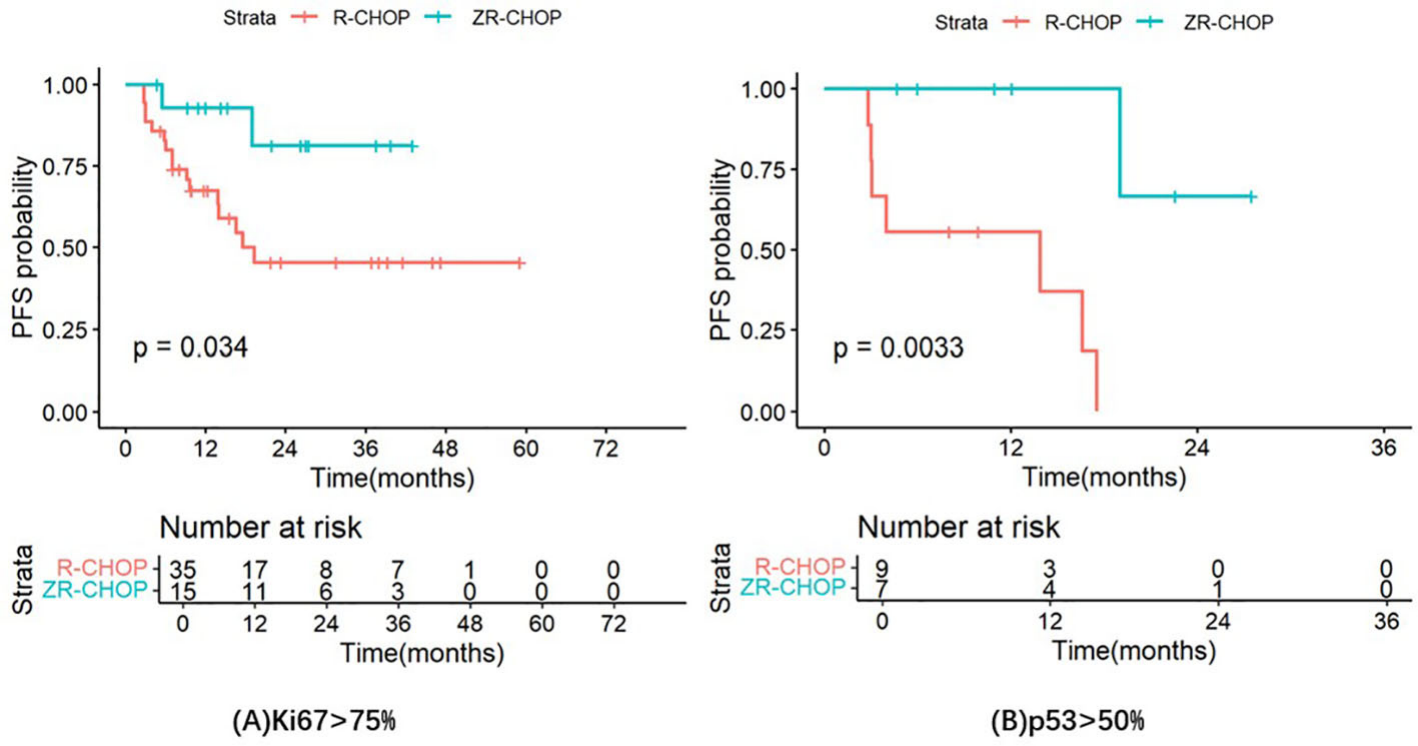
Comparisons of PFS in the Ki67 index >75% subgroup **(A)** and the p53 expression >50% subgroup **(B)** between the two treatment groups.

PFS was longer in the ZR-CHOP regimen group compared to the R-CHOP regimen group in several subgroups, including patients with age ≥60 (P= 0.12), Ann Arbor staging system III to IV (P= 0.17), IPI score of 3-5 (P= 0.23), elevated β2-MG (P= 0.33), elevated LDH (P= 0.29), extramedullary infiltration (P= 0.074), and non-GCB subtype (P= 0.33). However, these differences were not statistically significant. Notably, there were significant differences in the Ki67 index >75% subgroup (P= 0.034) and the p53 expression >50% subgroup (P= 0.0033) between the two treatment groups. These results are illustrated in **Figure 6**.

### Safety

Patients in both groups experienced comparable levels of adverse events (AEs), with hematological toxicities being the most prevalent side effect. **Table 2** provides a summary of the AEs reported in both groups, including hematological AEs, pulmonary infection, atrial fibrillation, hemorrhage, hyperuricemia, elevated creatinine, alanine aminotransferase (ALT) or aspartate aminotransferase (AST) elevation, nausea, apositia, diarrhea and fatigue. However, there was no statistical difference between the two groups.

**Table 2:** Adverse events in both groups.

Adverse events	R-CHOP(n=55)	ZR-CHOP(n=23)	P
Grade 3–4 anemia, n (%)	26 (47.3)	11 (47.8)	1
Grade 3–4 thrombocytopenia, n (%)	28 (50.9)	7 (30.4)	0.159
Grade 3–4 neutropenia, n (%)	30 (54.5)	9 (39.1)	0.321
Grade 3–4 febrile neutropenia, n (%)	13 (23.6)	6 (26.1)	1
Pulmonary infection, n (%)	17 (30.9)	9 (39.1)	0.661
Atrial fibrillation, n (%)	1 (1.8)	0 (0.0)	1
Hemorrhage, n (%)	4 (7.3)	1 (4.3)	1
Hyperuricemia, n (%)	19 (34.5)	4 (17.4)	0.214
Elevated creatinine, n (%)	5 (9.1)	0 (0.0)	0.323
ALT or AST elevation, n (%)	21 (38.2)	6 (26.1)	0.446
Nausea, n (%)	26 (47.3)	6 (26.1)	0.138
Apositia, n (%)	17 (30.9)	6 (26.1)	0.878
Diarrhea, n (%)	9 (16.4)	2 (8.7)	0.596
Fatigue, n (%)	32 (58.2)	8 (34.8)	0.102

ALT, Alanine Aminotransferase; AST, Aspartate Aminotransferase.

## Discussion

The new generation of BTK inhibitors, zanubrutinib, in the treatment of DE-DLBCL is worth exploring, and relevant prospective studies are currently in progress. Our study is the first to retrospectively compare the efficacy and safety of ZR-CHOP with R-CHOP for untreated DE-DLBCL. The results of this study showed a higher CR rate in the ZR-CHOP regimen group compared to the R-CHOP regimen group (4 cycles of treatment: 82.6% vs. 50.9%, P= 0.019; 6 cycles of treatment: 95% vs. 65.2%, P= 0.025). ORR was higher in the ZR-CHOP group than that in the R-CHOP group, but not statistically significant (95.7% vs. 89.1%, P= 0.624; 100% vs. 87%, P= 0.219). In the PHOENIX trial (25), the CRR was 64.9% in the DE-DLBCL patients treated with R-CHOP (similar to 65.2% in this study), and 67.5% in those treated with ibrutinib plus R-CHOP. In our study, the CRR of DE-DLBCL patients treated with ZR-CHOP was 95%, suggesting that zanubrutinib may have a better CRR than ibrutinib for DE-DLBCL. In the ALPINE trial (35), zanubrutinib performed better than ibrutinib in patients with relapsed/refractory CLL. ORR remained higher in zanubrutinib compared with ibrutinib (85.6% vs. 75.4%; RR: 1.13 [95% CI, 1.05-1.22]). The observed differences in efficacy between ibrutinib and zanubrutinib can be attributed to their distinct molecular structures. Firstly, zanubrutinib features a different ring system in the middle of its structure, altering the molecule’s planarity (36). Less planar structures often exhibit better solubility, which may explain why the plasma drug exposure of zanubrutinib is eight times greater than that of ibrutinib (37). Additionally, zanubrutinib shows a high and stable occupancy rate as a BTK inhibitor in peripheral blood mononuclear cells and lymph nodes (38). Moreover, zanubrutinib has a lower maximum half inhibitory concentration (IC50) compared to ibrutinib, indicating stronger inhibitory activity (39). Secondly, unlike ibrutinib, zanubrutinib lacks a pyrimidine ring (36). This absence reduces its ability to bind to kinases other than BTK, resulting in enhanced target selectivity and fewer adverse events. Consequently, the rate of treatment discontinuation due to adverse events or disease progression is lower for patients receiving zanubrutinib compared to those treated with ibrutinib (35). Therefore, the study of zanubrutinib in the treatment of DE-DLBCL deserves further exploration. Our study suggests that ZR-CHOP enables more DE-DLBCL patients to achieve CR, increasing the depth of remission.

In this study, ZR-CHOP regimen tended to prolong PFS and OS compared with R-CHOP regimen. The PFS rate of DE-DLBCL patients treated with ZR-CHOP was higher than that of R-CHOP, even though the difference was not significant (3-year PFS rates: 77.5% vs. 47.6%, P= 0.072). In addition, the PFS rate of R-CHOP in the treatment of DE-DLBCL (3-year PFS rates was 47.6%) was consistent with previous studies (5-year PFS rate was 44%; 5-year PFS rate was 46%) (11, 19). Similarly, the OS rate in the ZR-CHOP group tended to be higher than that in the R-CHOP group, but there was no significant statistical significance (3-year OS rates: 95.2% vs. 81.1%, P= 0.29). However, DE-DLBCL patients treated with R-CHOP had a higher OS rate (3-year OS rate was 81.1%) than in these studies (5-year OS rate was 39%; 5-year OS rate was 52%) (11, 19). The possible reasons for the difference in OS rate between our study and the prospective studies include the insufficient number of enrolled patients, the limited follow-up time, and the switch to other treatment after poor response to previous therapy. Therefore, ZR-CHOP has the potential to improve the prognosis of DE-DLBCL patients, especially in term of PFS.

It is clear that p53 overexpression (more than 50%) is associated with poor prognosis in DLBCL patients (5-year PFS of DLBCL with p53 expression >50% was 28%; 5-year OS of DLBCL with p53 expression >50% was 46%) (40–46). However, there is a lack of research data on the treatment response and progonosis of DE-DLBCL with strong p53 expression. In our study, the CR rates of DE-DLBCL patients with p53 expression >50% and p53 expression ≤50%, treated with R-CHOP, were 11.1% and 58.7% (P= 0.025), and the 1-year PFS rates were 55.6% and 74.8% (P= 0.00054), respectively. Therefore, DE-DLBCL with strong p53 expression had worse response and PFS than DLBCL with strong p53 expression. The CRR of DE-DLBCL patients with p53 expression >50% in the ZR-CHOP group was significantly higher than that in the R-CHOP group after 4 courses of treatment (85.7% vs. 11.1%; P= 0.013), but there was no statistical significance after 6 courses of treatment (85.7% vs. 50%; P= 0.565). In fact, some patients were switched to alternative medications due to poor treatment response, which led to a decrease in the number of patients on subsequent treatment and might diminish the difference between the two treatment groups after 6 courses. In addition, DE-DLBCL patients with p53 expression >50% had significantly longer PFS treated with ZR-CHOP than those treated with R-CHOP (1-year PFS rate: 100% vs. 55.6%, P= 0.0033). Therefore, the treatment response and prognosis of DE-DLBCL patients with p53 expression >50% are poorer; however, the ZR-CHOP regimen can assist these patients in achieving earlier CR and significantly enhancing both CRR and PFS.

Some studies suggest that high Ki67 index had significant adverse prognostic effects in DLBCL, but the specific cut-off value is not uniform at present (47–52). Salles et al. found that the OS of DLBCL patients with Ki67 index ≤75% was significantly longer than that of patients with Ki67 index >75% (P<0.05) (53). In a study of R-CHOP in DLBCL patients, elevated Ki67 index seems to be associated with inferior EFS in patients with DLBCL treated with R-CHOP (P= 0.012) (54). The CR rates of DLBCL patients with Ki67 ≥85% and Ki67 <85% were 69.6% and 81.6%, and the 2-year PFS rates were 44.3% and 74.1%, respectively. In our study, the CR rates of DE-DLBCL patients with Ki67 >75% and Ki67 ≤75%, receiving R-CHOP, were 64.3% and 66.7% (P= 1), and the 2-year PFS rates were 45.4% and 52.8% (P= 0.74), respectively. The data of the high Ki67 group are similar to the results of the above study (54), and the efficacy and prognosis of the high Ki67 group were worse than those of the low Ki67 group. In DE-DLBCL patients with Ki67 index ≤75%, there was no significant difference in CRR between the ZR-CHOP group and the R-CHOP group after 4 courses (75% vs. 50%; P= 0.432) or 6 courses of treatment (100% vs. 66.7%; P= 0.276). The CRR of DE-DLBCL patients with Ki67 index >75% in the ZR-CHOP group was significantly higher than that in the R-CHOP group after 4 courses of treatment (86.7% vs. 51.4%; P= 0.042), but there was no statistical significance after 6 courses of treatment (92.9% vs. 64.3%; P= 0.107). The potential reasons are analogous to those observed in the strong p53 expression group, including a limited sample size and treatment modifications in certain patients. DE-DLBCL patients with Ki67 index >75% treated with ZR-CHOP had significantly longer PFS than those treated with R-CHOP (1-year PFS rate: 92.9% vs. 67.5%, P= 0.034). Therefore, ZR-CHOP may have better efficacy and improve prognosis for DE-DLBCL with Ki67 >75%, and delay disease progression in this population.

The adverse events (AEs) associated with the ZR-CHOP and R-CHOP regimens were comparable. Hematologic toxicity was the predominant AE in the ZR-CHOP group, whereas pulmonary infections (39.1%) and fatigue (34.8%) were the most common non-hematologic AEs. Notably, there were no instances of atrial fibrillation and fewer hemorrhagic events (4.3%) in the ZR-CHOP group. Furthermore, the incidence of grade 3–4 neutropenia and grade 3–4 thrombocytopenia was lower in the ZR-CHOP group compared to that in the R-CHOP group. As the treatment of the ZR-CHOP group occurred over the past three years, a greater number of patients opted for certain costly medications to prevent AEs due to enhanced awareness regarding treatment options, such as thrombopoietin receptor agonists and pegylated recombinant human granulocyte colony-stimulating factor, which may contribute to reducing and mitigating AE occurrences in this population.

This is the first retrospective study to compare the efficacy and safety of ZR-CHOP and R-CHOP in the treatment of DE-DLBCL. Compared with R-CHOP, zanubrutinib plus R-CHOP effectively increased the CR rate of DE-DLBCL and showed a tendency to delay disease progression, especially in DE-DLBCL with Ki67 index >75% or p53 expression >50%. These evidences support zanubrutinib plus R-CHOP as a first-line treatment option for untreated DE-DLBCL. This study has the limitations of small number of cases and short follow-up time, so large-scale prospective clinical studies are needed to further confirm the efficacy and safety of ZR-CHOP in DE-DLBCL.

## Data Availability

The raw data supporting the conclusions of this article will be made available by the authors, without undue reservation.
